# The Connection Between Associative Memory and Semantic Similarity: Evidence From Fan Experiments and Distributional Models

**DOI:** 10.1111/cogs.70210

**Published:** 2026-04-26

**Authors:** Philine Link, Diego Frassinelli, Leendert van Maanen, Jakub Dotlačil

**Affiliations:** ^1^ Institute for Language Sciences Utrecht University; ^2^ Center for Information and Language Processing LMU Munich; ^3^ Department of Experimental Psychology Utrecht University

**Keywords:** Rational analysis of memory, Fan effect, Spreading activation, Vector‐based models of meaning, Skip‐gram models, Memory retrieval, Associative memory

## Abstract

Memory retrieval is prone to interference: when multiple concepts in memory match a given retrieval cue, recall becomes slower and less accurate. This has repeatedly been studied in fan effect experiments in which participants learn facts that are combinations of person–location pairs. These experiments manipulate the fan of a concept—the number of facts linked to it—establishing interference. The standard theoretical account invokes spreading activation: when a cue is linked to multiple memory traces, activation spreads across them, reducing the target's retrievability. We study whether this spreading activation is triggered only by explicitly learned associations or also by semantic similarity. We show that spreading activation in the rational analysis of memory is pointwise mutual information and that similarity in at least some vector‐space models of meaning approximates the same quantity, which makes such models potentially formal implementations of the rational analysis of memory. In two behavioral experiments using Dutch‐language stimuli, we first replicate the classical fan effect. Experiment 2 tests whether this interference effect can be elicited through semantic similarity alone, using pretrained word embeddings to construct semantic fans. We find that items in higher semantic‐fan conditions are retrieved more slowly and less accurately, mirroring patterns from Experiment 1. In a simulation, we show that similarity in embedding spaces predicts retrieval difficulty in a manner consistent with rational models of memory. Together, these results formally connect vector‐space models of meaning with the rational analysis of memory, and demonstrate that semantic similarity is sufficient to produce associative interference in memory.

## 1 Introduction

A robust finding in cognitive science is that memory retrieval slows down and becomes less accurate when several items in memory share features with the retrieval target. These features function as retrieval cues. When multiple items match the same cue, they become activated alongside the target, reducing the accessibility of the target. Such similarity‐based interference has been observed across domains such as sentence processing (e.g., Jäger, Engelmann, & Vasishth, 2017; Lewis & Vasishth, 2005; Van Dyke, 2007; Van Dyke & McElree, 2011), visual working memory (Oberauer & Lin, 2017), and episodic memory, most prominently in the fan effect (Anderson, 1974; Anderson & Reder, 1999). Focusing on the latter, fan effect studies show that increasing the number of concepts associated with a cue leads to increased response times (RTs) and lower accuracy. A common theoretical explanation of the fan effect and other similarity‐based interference findings is based on spreading activation (Collins & Loftus, 1975). Activation refers to the accessibility of a memory representation (Anderson & Reder, 1999). When multiple items share cues, activation spreads across them, producing the typical interference pattern. This raises the broader question about whether different kinds of similarity, that is, shared cues, elicit the same retrieval dynamics.

Building on this idea, multiple studies have explored how memory performance is affected by the similarity of words, quantified using semantic representations from high‐dimensional vector spaces (e.g., Griffiths, Steyvers, & Tenenbaum, 2007; Jones, Kintsch, & Mewhort, 2006; Mandera, Keuleers, & Brysbaert, 2017; Meghdadi, Duff, & Demberg, 2026; Osth, Shabahang, Mewhort, & Heathcote, 2020; Reid & Jamieson, 2023; Smith & Vasishth, 2020). Several recent frameworks have further combined representational and process‐level approaches to memory by including semantic, orthographic, and phonological information (Guitard, Saint‐Aubin, Reid, & Jamieson, 2025), and incorporating perceptual information of words (e.g., letter position in Osth & Zhang, 2024). The success of these models in predicting retrieval behavior in humans can be seen as support for the correspondence between similarity in vector spaces and spreading activation in models of human memory. When a cue is represented as a vector, its similarity to neighboring vectors determines how strongly these concepts become activated. This mirrors retrieval dynamics proposed in models of human memory.

In this paper, we focus on the explanation of similarity‐based interference grounded in the rational analysis of memory (Anderson, 1990; Anderson, 1991), a framework which assumes that memory processes are adapted to the structure of the environment and the goals of the user. This has been implemented in the Adaptive Control of Thinking‐Rational cognitive architecture (ACT‐R, Anderson, Bothell, & Byrne, 2004; Anderson, 2007), which provides a computational model of how memory operates under these rational principles.

The rational analysis of memory, as implemented in ACT‐R, correctly predicts the fan effect (Anderson & Lebiere, 1998; Anderson & Reder, 1999), and it has also been at least partially successfully applied to data other than reaction times and accuracies, such as data from electroencephalography (EEG, Borst, Schneider, Walsh, & Anderson, 2013). It has been hypothesized that the rational analysis of memory model and models of word meaning are closely related (Günther, Rinaldi, & Marelli, 2019; Hollis, 2017). However, there is currently no explicit link between the two approaches. That is, we miss a formal account that directly maps semantic similarity from vector‐space models onto rational analysis of memory, which limits our understanding of spreading activation, on the one hand, and the relation of vector‐space models to memory, on the other hand. One concrete limitation for the studies on spreading activation is that pre‐existent associations are difficult to incorporate into the ACT‐R framework, which does not take exposure to naturalistic language into account. Unlike distributional models that determine associations based on large corpora, ACT‐R cannot straightforwardly capture the kinds of associations formed in every day communication or over longer discourse.

In this paper, we provide the missing formal link. More concretely, we link spreading activation to skip‐gram models with negative sampling, which are one of the most popular and highly used classes of vector‐space models of lexical meaning. Using these models, we, furthermore, show that the fan effect, and, therefore, the spread of activation in memory, can be extended to semantic relationships inferred from natural language use.

We start by establishing a link between the rational analysis of memory, spreading activation, and vector‐space models of meaning. We then move on to experimental evidence, presenting two experiments investigating the fan effect in Dutch. Experiment 1 replicates the classic fan paradigm (Anderson, 1974; Anderson & Reder, 1999). We discuss the experiment for two reasons. First, it is important to establish that Dutch shows classical fan effects, since the second experiment is run on Dutch as well. Second, classical fan experiments tested the fan effect when participants were explicitly instructed to memorize the fan of words by listing all associations to a given probe. This makes the differences in fan size between conditions clearly visible. We wanted to confirm that the results are not driven by participants' explicit awareness of the fan size during learning. Experiment 2 is the main empirical contribution in this paper. It extends the fan‐effect design by replacing repeated identical concepts with unique semantically similar stimuli derived from vector‐space models to create the associative fan. Experiment 1 and previous studies using the fan effect can be seen as cases in which semantic similarity is maximal. That is, words within the same fan are identical. Experiment 2 tests whether the fan effect persists when the fan is defined by high but not perfect semantic similarity. Finding the fan effect in such a setup provides novel empirical support for the connection between the vector‐space models of meaning and the rational models of memory. We conclude by showing modeling results supporting the link between vector‐space models and the rational analysis of memory. As we will see, under the linking hypothesis, the vector‐space models predict fan effects, both the classical ones and the ones we establish in Experiment 2.

## 2 Predicting fan effects in the rational analysis of memory and vector‐space models of meaning

In the rational analysis of memory (Anderson, 1990; Anderson, 1991), the activation of a piece of information (a chunk) represents the model's estimation of the probability that the fact is needed. The activation is dependent on two factors: the history factor, which represents the activation due to the past usages of the chunk, and the context factor, which models how the cues in the context affect the activation of the chunk. For associations, including the fan effects, the context is the crucial factor (Anderson, 1991; Anderson & Reder, 1999), and the history factor can be ignored. Following the terminology in ACT‐R, we call the activation due to context the spreading activation. For some cue j and piece of information i, the formula for the spreading activation $S_{ji}$ is shown in (1), where P(i) is the probability that the chunk i is needed and P(i|j) is the probability that i is needed given that we received a cue, j.

(1) $S_{ji}=\log\frac{P(i|j)}{P(i)}$

Defined this way, spreading activation produces a fan effect (Anderson & Lebiere, 1998). This can be appreciated by observing that P(i|j) is inversely proportional to the fan of a word. This is because a higher fan—more associations to a cue—decreases the probability that a specific chunk i is needed. A lower spreading activation is translated to more errors and slower responses (cf. Anderson, 2007).

The rational analysis formula for spreading activation, given in (1), is pointwise mutual information (pmi) (Brasoveanu & Dotlačil, 2020, Chapter 6; Farahat, Pirolli, & Markova, 2004; Stanley & Byrne, 2016). The pmi metric is often used in computational linguistics to measure associations between terms (see Levy & Goldberg 2014 for discussion). This is crucial for us in establishing a link between the rational analysis of memory and vector‐space models.

The underlying interpretation of spreading activation as the pmi measure makes it straightforward to link the rational analysis of memory and count distributional semantic models that use pmi to weigh the co‐occurrences. In this paper, however, we focus on models that superseded count models, which are so‐called context‐predicting semantic‐vector models (see Baroni, Dinu, & Kruszewski 2014 for a comparison of counting vs. predicting models). The vector meaning space of those models is constructed through training to optimally predict the probability with which a word would appear in a particular context. To make this concrete, our focus will be on skip‐gram with negative sampling (Mikolov, Sutskever, Chen, Corrado, & Dean, 2013).

Skip‐gram models predict the probability that a pair of words, labeled as the target word, t, and the context word, c, co‐occur. During training, skip‐gram models maximize the probability of that t and c co‐occur, and minimize this probability for “negative” samples: these are (t,c) pairs such that for the target word t, a word c is selected at random from the vocabulary. This learning objective connects skip‐grams and the pmi measure (Levy & Goldberg, 2014): Under the assumption that a meaning representation is independent from all others, the similarity of the meaning representation of t and c (technically, the dot product of their meaning vectors) approximates the pmi measure of the two elements shifted by log(k), where k is the number of negative samples taken in the training.

Thus, we see that the spreading activation in the rational analysis of memory and the dot‐product of word vector representations of skip‐gram models are underlying the same mathematical concept, the pmi measure. Consequently, if cognitive scientists see the ACT‐R memory module—as an implementation of the rational analysis—as a representation of human memory, it gives reason to consider the skip‐gram model as another such representation, at least with respect to associative structure. The main difference between the ACT‐R spreading activation and the skip‐gram model are their applications: the former is used as a snapshot of concepts in the episodic memory, while the latter is a model of language, in our case, context‐target word predictions.

If there is a link between spreading activation in models of human memory and semantic similarity in vector‐space models, then we should be able to find fan effects based on semantic similarity, in addition to fan effects based on word identity.

We start with the experiment that establishes that fan effects can be observed in Dutch, because we use Dutch expressions in Experiment 2. Along the way, we also show that the fan effect holds even if participants are not explicitly shown the fan size of individual words—which is predicted by the rational analysis of memory, but to the best of our knowledge, has not been tested before.

The second experiment is the main result of this paper: it tests the formal link between the activation in the rational analysis of memory and skip‐gram vector‐space models in ways that, to the best of our knowledge, have not been established so far. In particular, we study how pretrained vector space can be leveraged to uncover memory association in fan‐effect experiments.

The experiment uses pretrained skip‐gram embeddings and combines them with the fan experiment in which semantically related words (according to the model) form a fan. If vector space models are good approximations of spreading activation, we expect that using their pretrained internal representation should also reveal a fan effect. The existence of such a fan effect has not been studied or even considered before, as far as we know. We will see that the experiment indeed shows the expected fan effect, supporting the just established link between vector‐space models of words and the rational analysis of memory.

## 3 Experiment 1—Replication study

The fan effect has previously been studied using experimental paradigms in which participants learn simple facts, for example, *The hippie is in the park* (Anderson & Reder, 1999). In these studies, individual concepts, in this case *hippie* and *park*, appear in multiple facts in combination with other concepts, creating an associative fan—the number of different facts linked to a given concept. After learning a set of facts, participants perform a retrieval task in which they determine whether a presented fact was previously studied (target) or not (foil).

Our first experiment was a modified replication of Anderson and Reder's (1999) fan experiment. In this replication, we tested the use of Dutch instead of English stimuli, as well as an adjusted learning method, which is described in detail below. Testing both of these changes served as a validation of the method for Experiment 2. We limited the experiment to two conditions, fan 2 and fan 4, and reduced the size of the stimulus sets to 24 target sentences (as compared to 48 sentences in Anderson & Reder, 1999) to shorten the experiment. Ethical approval was obtained from the local ethics committee at the Institute for Language Sciences at Utrecht University.

### 3.1 Methods

We conducted a fan experiment using short sentences that combined person and location words following the pattern “De *persoon* is in de/het *locatie” (“The person is in the location”)*. While one concept was fixed at fan 2 (person or location), the other concept varied between fan 2 and fan 4 (location or person). For each participant, we created a unique stimulus set of 24 sentences. In eight of the stimulus sentences, both concepts were set to fan 2 (2–2) and in the remaining 16 sentences, either the person concepts (4–2) or the location concepts (2–4) were set to fan 4. Half of the participants were tested on a stimulus set, which varied person fan between 2 and 4, while location fan was kept at fan 2, and the other half on a set that varied the location fan. An example of the pattern according to which the concepts were combined is shown in Table 1.

**Table 1:** Example of stimuli used in Experiment 1

Person	Location	Example sentences
a	A	*De kapitein is in de toren*
b		*De verkoper is in de toren*
a	B	*De kapitein is in het zwembad*
c		*De soldaat is in het zwembad*
…		
e	E	*De schilder is in de grot*
f		*De cowboy is in de grot*
e	F	*De schilder is in het dorp*
f		*De cowboy is in het dorp*
e	G	*De schilder is in de schuur*
g		*De rechter is in de schuur*
e	H	*De schilder is in het ravijn*
g		*De rechter is in het ravijn*
…		
Repaired foils:		*De schilder is in de toren*
		*De schilder is in het zwembad*

*Note*: Here, the lowercase letter refers to a person item (e.g., a = *kapitein*, captain), while the uppercase letter refers to a location item (e.g., A = *toren*, tower). In this example, person fan is varied (a = fan 2, e = fan 4), while location fan remains at fan 2. The foil sentences were created with new pairings of lexical items that were used in the target sentences.

#### 3.1.1 Materials

To create the stimulus sets, we used common Dutch words describing occupations for person words and spaces that could be combined with the preposition *in* for location words. To control for effects of word length and complexity on RTs, all selected words were morphologically simple and between four and nine characters long. Additionally, we matched the frequency of items within each category (person or location) according to the Zipf scale based on the SUBTLEX‐NL corpus (Keuleers, Brysbaert, & New, 2010) for person words (mean = 4.14, range = 3.27–5.38) and location words (mean = 4.20, range = 3.03–5.09). All sentences followed the previously described pattern “De *persoon* is in de/het *locatie*,” where we replace *persoon* and *locatie* with the respective stimulus word. We balanced the number of sentences containing *de* and *het* as the determiner of the location within stimulus sets. Each participant received a unique stimulus set, which was created according to the pattern in Table 1 by randomly assigning lexical items from the person and location word lists to the letters.

#### 3.1.2 Participants

We collected data in the lab (*N* = 50) and online (*N* = 100). For the lab experiment, we recruited participants through the ILS labs database and the SONA system of the faculty of Social and Behavioral Sciences at Utrecht University. All participants gave informed consent. For the online experiment, we recruited participants through Prolific (all had high approval ratings, 95–100%). All participants were adult Dutch L1 speakers from the Netherlands.

#### 3.1.3 Design and procedure

The experiment was programmed in PsychoPy (Peirce et al., 2019). It took approximately 45 min to complete and consisted of three parts reflecting the structure of classic fan experiments: an exposure phase, a learning phase, and a testing phase.

**Exposure and learning phases**

In the exposure phase, each sentence was presented once. Participants were instructed to read each sentence carefully and continue to the next sentence via button press. For the subsequent learning phase, we created two questions for each sentence: “Waar is de *persoon*?” (“Where is the *person*?”) and “Wie is in de/het *locatie*?” (“Who is in the *location*?”), replacing *persoon* and *locatie* with the words used in the stimulus sentences. The sentence “De kapitein is in de toren” would, therefore, be studied with the questions “Waar is de kapitein?” and “Wie is in de toren?,” resulting in 48 questions for 24 stimulus sentences. The questions were presented in randomized order with four response options. One response option was correct, while the other responses were randomly sampled from the stimulus set. The incorrect responses were items that were not part of the fan in question. Participants responded to the questions by clicking a response option. There was no time limit for the response. If a question was answered correctly, it was dropped for the current round of the learning phase, if not, it was repeated at the end of the round. After each response, participants saw the sentence the questions referred to and whether their response was correct or not. One round of the learning phase was completed once all questions had been answered correctly. Every participant completed two rounds.

The learning phase used in this experiment differed from previous fan experiments (e.g., Anderson, 1974; Anderson & Reder, 1999; Bunting, Conway, & Heitz, 2004; Cantor & Engle, 1993). In previous experiments, participants were required to list all possible locations in response to a requested person and vice versa. This setup in the learning phase potentially makes the fan manipulation clear to participants by requiring them to list either two or four associations depending on the fan condition. To address this potential confound, we used a method that probed for one association per learning trial by presenting it among four possible response options to the given cue. We aimed to verify that the fan effect persists under this methodological change, ensuring that our results are not driven by participants' explicit awareness of the fan size during learning.

**Testing phase**

After the learning phase, participants completed three rounds of the testing phase. In this part of the experiment, participants saw a sentence on the screen and were instructed to respond via button press whether they had learned the sentence in previous phases (targets) or not (foils). The mapping of response keys was counterbalanced between participants and there was no time limit for the response. Participants were instructed to respond as quickly and accurately as possible, as is common in many behavioral experiments. In each round of the testing phase, participants responded to a total of 48 sentences (24 target sentences and 24 foils). After every 16 responses, participants could take a break.

### 3.2 Results

From both data collections of the first experiment, we excluded participants with a mean accuracy <0.6 in the testing phase to exclude responses given at chance. We chose this threshold to ensure that participants performed reliably above chance while still allowing some variability. This resulted in 46 final participants in the lab and 88 in the online data set. We analyzed accuracy and RT data in R (R Core Team, 2024) with hierarchical Bayesian generalized linear mixed models for both data sets separately using the brms package (Bürkner, 2017).

#### 3.2.1 Accuracy

Table 2 presents the descriptive summary of mean accuracies recorded during the testing phase. In both the lab and online data sets, accuracy decreased for experimental stimuli with a location fan of 4 compared to those with a location fan of 2, indicating that participants made more retrieval errors when the location fan was higher. A similar pattern was observed for stimuli with a higher person fan in the online data. In the lab data, the accuracy for the condition with a person fan of 2 did not differ from the fan 4 condition. This could be due to the smaller sample size in the lab data (*N* = 46) and the difference in data collection methods (online experiment vs. lab experiment). For a better estimate of the effect size, we merged the data from both data collections (*Accuracy merged*).

**Table 2:** Descriptive summary of mean accuracies with standard errors (SE) in the lab and online data collections of Experiment 1, as well as the merged data set

Person fan	Location fan	Accuracy lab (SE)	Accuracy online (SE)	Accuracy merged (SE)
2	2	0.85 (0.008)	0.82 (0.006)	0.83 (0.005)
2	4	0.80 (0.008)	0.77 (0.007)	0.78 (0.005)
4	2	0.86 (0.008)	0.76 (0.007)	0.79 (0.005)

We fitted the same hierarchical Bayesian model with a Bernoulli likelihood and a logit link function to the accuracy data from both data collections separately, as well as on the merged data. The model was fitted with four sampling chains, each running for 3000 iterations. The first 1500 sampling iterations of each chain were discarded as warm‐up. The prior distribution of the intercept was normally distributed with μ = 0 and σ = 2. For the slopes, we used a normal distribution with μ = 0 and σ = 1. The standard deviations of the random effects received a prior with a normal distribution with μ = 0 and σ = 1. Lastly, the LKJ distribution (Lewandowski, Kurowicka, & Joe, 2009) was used for the random effects correlation with η = 2. The model converged. The reported Rhat values of the model were all close to 1 (max. Rhat = 1.004), indicating that the chains mixed well, and no divergent transitions were reported after warm‐up.

In the nested model, the independent variables were fixed effects for foils and targets (*fan_foil*, *fan_target*) that combine the stimulus type (foil or target) and the fan size (2 or 4) as well as a fixed effect *stimulus_type*, which describes whether a stimulus was a target or a foil. The variable *fan_foil* was sum‐contrast coded: when a foil sentence had a fan of 2, *fan_foil* was coded as −1, when it had a fan of 4, it was 1. If the row referred to a target sentence, *fan_foil* was coded 0. The same pattern applied to the variable *fan_target*. The variable *stimulus_type* was coded as −1 for foil sentences and 1 for target sentences. Random intercepts and slopes for each participant (*pp_num*) and word (*fan_word*) were also included, producing the following model:

(2) $\begin{array}{ccc} \text{correct} & ∼ & 1+\text{fan}\_\text{foil}+\text{fan}\_\text{target}+\text{stimulus}\_\text{type}\\ & & +\;(1+\text{fan}\_\text{foil}+\text{fan}\_\text{target}+\text{stimulus}\_\text{type}|\text{pp}\_\text{num})\\ & & +\;(1+\text{fan}\_\text{foil}+\text{fan}\_\text{target}+\text{stimulus}\_\text{type}|\text{fan}\_\text{word}) \end{array}$

**Fig. 1:**
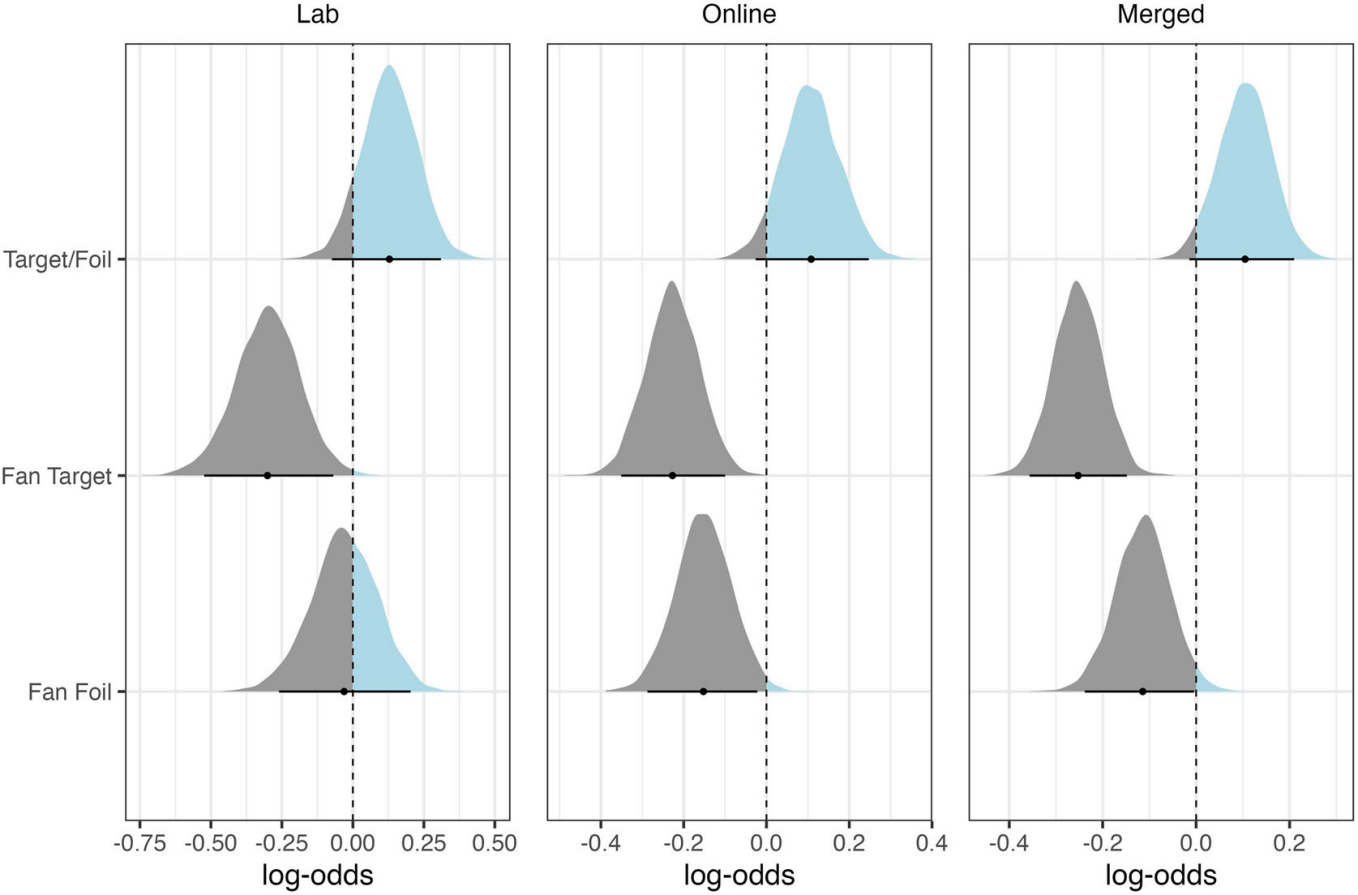
Posterior distributions of the accuracy model for lab data (left), online data (middle), and merged data (right).

The resulting posterior distributions of fitting the accuracy model to both the lab and the online data as well as the merged data can be seen in Fig. 1. To analyze the merged data, we added a sum‐contrast coded fixed effect for the type of data collection to (2). The distributions represent the range of possible values for each predictor, with the 95% credible intervals (CIs) marked by the horizontal lines. As shown, the posterior for Target/Foil (*stimulus_type*) is largely shifted toward positive log‐odds, whereas the posterior distribution for Fan Target (*fan_target*) is almost exclusively negative. A shift toward negative log‐odds means that as the fan increases (from fan 2 to fan 4), participants' accuracy on the retrieval task decreases. This trend can also be observed for the posterior distribution of Fan Foil (*fan_foil*) in the online data. For the lab data, the distribution of Fan Foil centers around zero, which indicates that there is little effect of an increase in fan size on the foils. This may be due to the smaller sample collected in the lab. Lastly, the posterior distribution for log‐odds in Target/Foil (*stimulus_type*) is shifted toward positive values, which shows that participants were more accurate in identifying target sentences compared to foils. However, since the 95% CIs include 0 in both data sets, this effect is uncertain.

#### 3.2.2 Reaction times

Before fitting a Bayesian linear mixed model or computing the means and SEs on the RT data, we removed extreme RTs faster than 200 ms and slower than 1 min and 30 s resulting in the removal of 223 data points from the online data set (1.03%). The RTs of the lab data set were not affected by this threshold. After this, we removed RTs exceeding three standard deviations from the mean. The descriptive summary of RTs collected in the testing phase of the lab and the online experiment as well as the merged data is shown in Table 3. The table shows that RTs for fan 4 are generally longer than RTs for fan 2. However, these effects were small for the person condition in the lab data and for the location condition in the online data. The values for the fan 2 condition appear identical across the person, location, and combined columns. This arises from the experimental design where one category was held at fan 2, while the other category varied between fan 2 and fan 4. Thus, fan 2 refers to trials in which both fan person and fan location were 2. As a result, the subset of trials corresponding to fan 2 is identical across person and location, leading to identical descriptive statistics.

**Table 3:** Descriptive summary of mean reaction times (in ms) with standard errors (SE) in the lab and online data collections of Experiment 1, as well as the merged data from both data collections

Condition	Fan	RT lab (SE)	RT online (SE)	RT merged (SE)
person	2	1736 (33.0)	1859 (27.8)	1815 (21.4)
	4	1746 (32.5)	2000 (30.9)	1917 (23.5)
location	2	1736 (33.0)	1859 (27.8)	1815 (21.4)
	4	1793 (34.4)	1862 (28.5)	1837 (21.9)
combined	2	1736 (33.0)	1859 (27.8)	1815 (21.4)
	4	1770 (23.6)	1932 (21.1)	1877 (16.1)

We analyzed RTs using a hierarchical Bayesian model with a shifted log‐normal likelihood, fitted separately for each data set. Other than the change in the dependent variable and likelihood, the RT model has the same structure as the accuracy model. The independent variables of the model were fixed effects for fan size combined with the condition (*fan_foil* and *fan_target*) as well as for the condition alone, that is, Target/Foil (*stimulus_type*). These fixed effects were sum‐contrast coded in the same way as in the accuracy model. The model also included by‐participant (*pp_num*) and by‐item (*fan_word*) random intercepts and slopes. We ran the model for 6000 iterations, the first 3000 of which were discarded as warm‐up, with four sampling chains. The intercept had a normal prior distribution with μ = 0 and σ = 15. The priors for the fixed effect coefficients were also normally distributed with μ = 0 and σ = 2. For the residual standard deviation and the standard deviation of random effects, we used normally distributed prior distributions with μ = 0 and σ = 2. Lastly, we used the LKJ distribution for the correlation of random effects with η = 2. The model converged with a maximum Rhat of 1.002, indicating that the chains mixed well and no divergent transitions were reported after warm‐up. We fit the following model with fan contrasts nested in target and foil conditions:

(3) $\begin{array}{ccc} \text{RT} & ∼ & 1+\text{fan}\_\text{foil}+\text{fan}\_\text{target}+\text{stimulus}\_\text{type}\\ & & +\;(1+\text{fan}\_\text{foil}+\text{fan}\_\text{target}+\text{stimulus}\_\text{type}|\text{pp}\_\text{num})\\ & & +\;(1+\text{fan}\_\text{foil}+\text{fan}\_\text{target}+\text{stimulus}\_\text{type}|\text{fan}\_\text{word}) \end{array}$

**Fig. 2:**
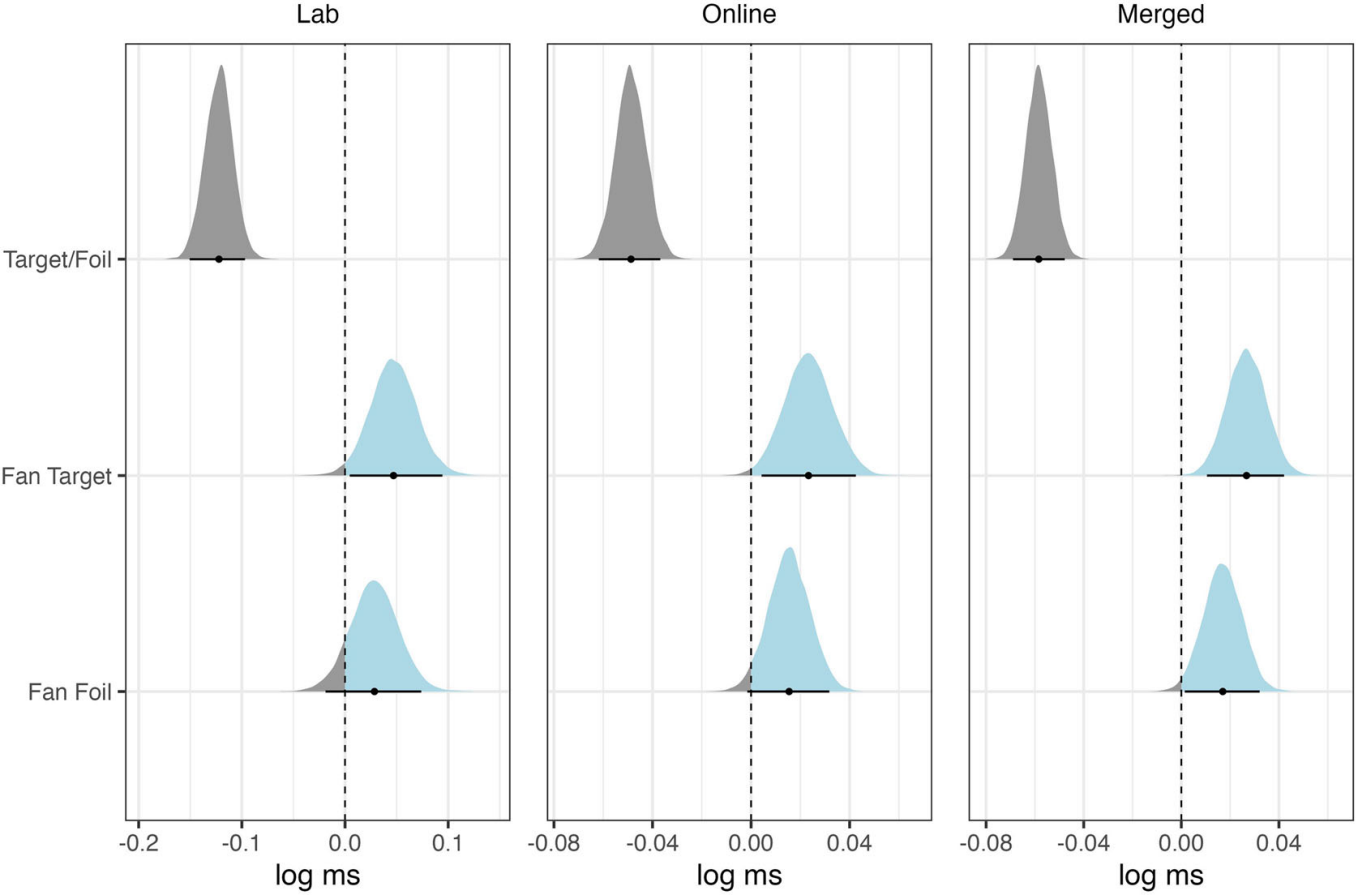
Posterior distributions of the reaction time model for lab (left) and online (middle) data, as well as merged data (right).

To analyze the merged data sets, we used the same model as in (3) but added a sum‐contrast coded fixed effect for the type of data collection. We increased the number of iterations to 8000 with a warm‐up of 4000 iterations to improve model convergence and achieve sufficient effective sample sizes across parameters. The posterior distributions of the RT model are shown in Fig. 2. The 95% credible intervals are again shown by the horizontal lines and the dot in the middle of the distribution is the mean of the estimate. In both the lab and the online data collection plots, the posterior distributions for Fan Target and Fan Foil are largely positive, indicating that reaction times in the retrieval task increase with an increase in fan for both target and foil conditions. As before, this tendency is more pronounced in the posterior distributions of the model fitted on online data and the merged data set, potentially due to the larger sample size. The distribution of the posterior estimates for Target/Foil are overall negative, reflecting longer reaction times in response to foils given that targets are coded as 1 and foils are coded as −1. Since the analyzed data contain only the correct response, this shows that targets are accepted more quickly than foils are rejected.^1^

To precisely quantify evidence for the fan effect in the target stimuli, we run a Bayes factors analysis on our data. Recall that Bayes factors are odds of marginal likelihood of two models, that is, they reveal how likely one model could have generated the data compared to the other model (see Schad, Nicenboim, Bürkner, Betancourt, & Vasishth, 2023; Wagenmakers, Lodewyckx, Kuriyal, & Grasman, 2010). We compare the M_1_ model provided above in (3), but since the Bayes factor is run on the merged dataset, M_1_ also includes the *data_collection* variable. The M_0_ model, which forms the baseline for the comparison, is identical to M_1_ except that the fixed effect *fan_target* is absent, see (4).

(4) $\begin{array}{ccc} M_{0}:\text{RT} & ∼ & 1+\text{fan}\_\text{foil}+\text{stimulus}\_\text{type}+\text{data}\_\text{collection}+\\ & & +\;(1+\text{fan}\_\text{foil}+\text{fan}\_\text{target}+\text{stimulus}\_\text{type}|\text{pp}\_\text{num})\\ & & +\;(1+\text{fan}\_\text{foil}+\text{fan}\_\text{target}+\text{stimulus}\_\text{type}|\text{fan}\_\text{word}) \end{array}$

The models share the same prior structures for all shared parameters. For *fan_target* in M1, we assume prior HalfNormal(0, 0.025). This is a constrained prior, carrying the assumption that the fan effect of target should be positive (i.e., higher fan increases RTs) but of small size (0.025 in log‐ms corresponds to the effect of 81 ms at the model intercept). Recall that constrained priors are needed for the correct application of Bayes factors, since we are exploring how likely data could be generated by the assumed model, and a comparison between two models would hardly be revealing if M1 had unrealistic priors for the crucial parameter of interest (see Schad et al., 2023 for discussion).

The Bayes factor (BF) analysis is done on models which were created collecting 20,000 samples on four chains (10,000 warm‐ups) using the Savage–Dickey ratio method. We find that ${\text{BF}}_{10}$ is 35.5. ${\text{BF}}_{10}$ expresses the odds of M_1_ over M_0_, therefore, values higher than 1 indicate relative evidence in favor of the M_1_ model. Using the interpretation of Bayes factor values provided by Jeffreys (1998), we conclude that we have very strong evidence for the model with the *fan_target* fixed effect, relative to the model without this fixed effect.

### 3.3 Discussion

We find the fan effect in both accuracy and RT data in Experiment 1, where we observed a decrease in accuracy and an increase in RTs for experimental items with a larger fan size. We, therefore, successfully replicated the fan effect in Dutch and with a learning method that does not give participants instructed knowledge of the fan of a learned item. This enables us to use the same experimental paradigm for the following experiment, which includes a semantic similarity manipulation.

## 4 Experiment 2—Semantic similarity manipulation

Based on the validation of the experimental methods in Experiment 1, we created the second experiment using the same design. However, in this experiment, the created associative fan was not due to the repetition of the lexical items in different contexts. Instead, we used pretrained embeddings from vector‐space models and selected semantically related items to create the fan. If our observation that skip‐gram models can represent a spreading‐activation memory model is correct, we would expect that pretrained embeddings could be used to induce fan effects. This would indicate that activation spreads between semantically similar items in memory and would further support our linking hypothesis between the rational analysis of memory and vector‐space models of meaning.

### 4.1 Methods

#### 4.1.1 Materials

**Fig. 3:**
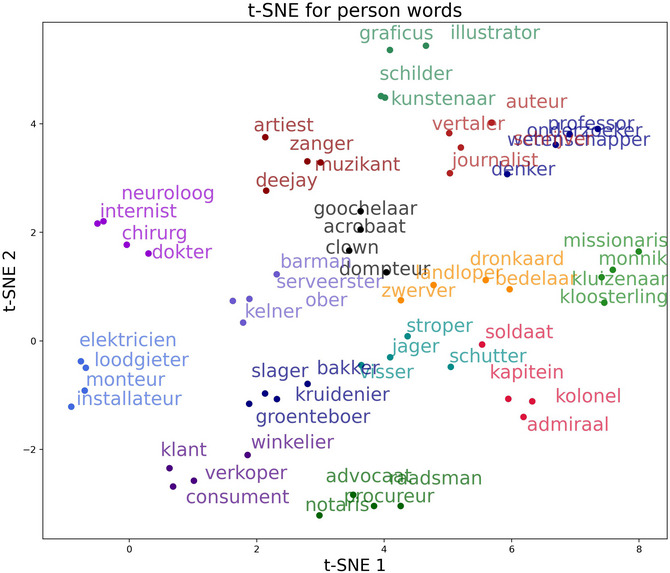
t‐SNE visualization of the word embeddings of the selected semantically similar stimuli of the *person* category. Words of the same color belong to the same group of semantically similar stimuli. Most selected groups cluster together neatly, showing that the word embeddings are similar.

**Fig. 4:**
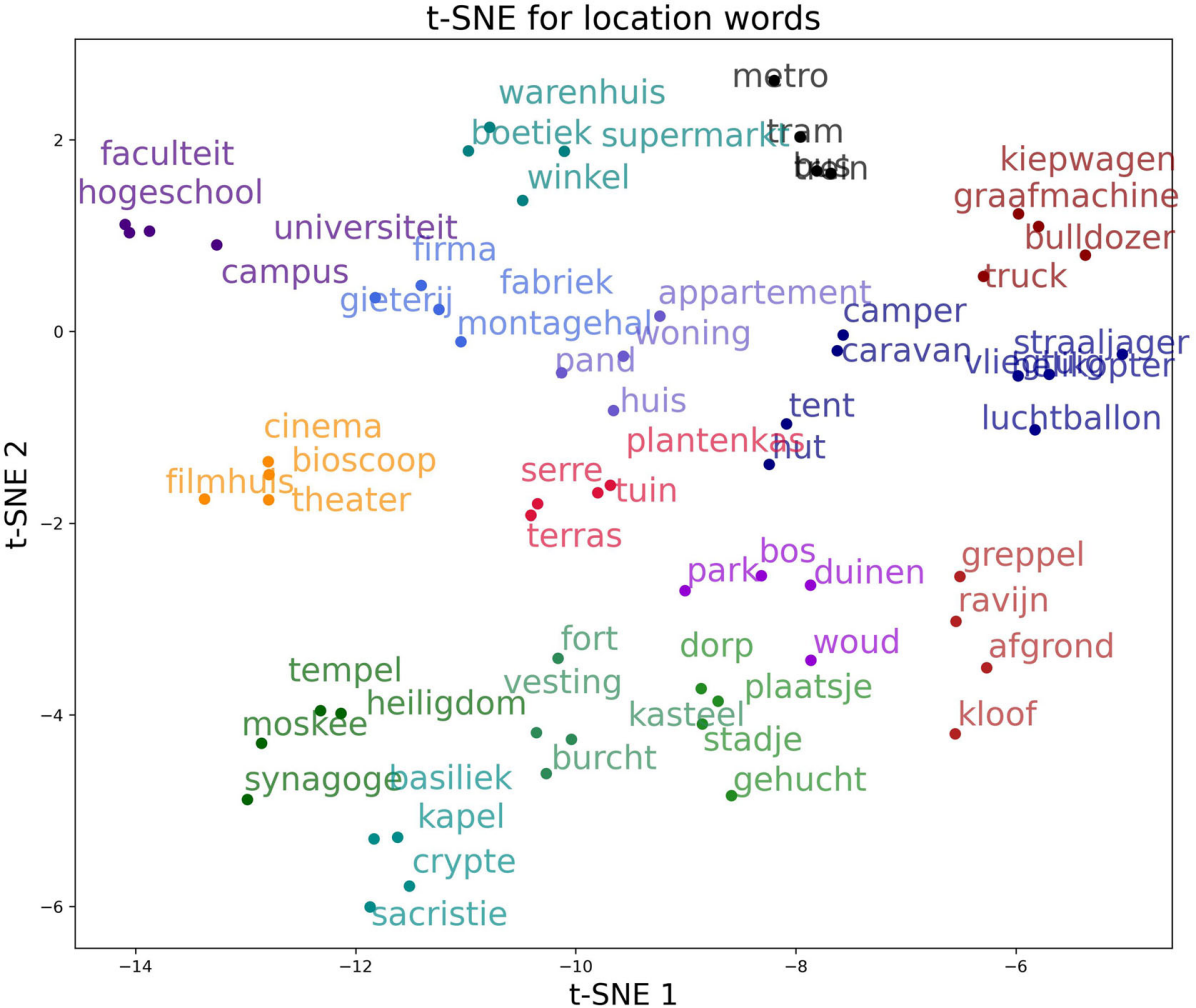
t‐SNE visualization of the word embeddings for the *location* category. Again, words of the same color belong to the same group of stimuli selected to be semantically similar to each other but distinct from other groups.

To create lists of semantically related items, we used the items from Experiment 1 and retrieved the 20 nearest neighbors to each of them from different fastText and Word2vec models. We initially compared the nearest neighbors of eight different vector space models but eventually built the lists of semantically related items based on two different fastText models by Grave, Bojanowski, Gupta, Joulin, & Mikolov (2018, CBOW) and Bojanowski, Grave, Joulin, & Mikolov (2017, skip‐gram), as well as the Word2vec model by Fares, Kutuzov, Oepen, & Velldal (2017, skip‐gram). All three vector space models were pretrained on Dutch corpora. These models were selected because within the 20 nearest neighbors, they provided candidates which fit within our selection criteria (person and location words) and which were judged by native speakers as close matches to the initial query word. We created 15 lists of four semantically similar items for the person and 16 lists for the location category by selecting items from the generated lists of neighbors that were similar to the meaning and category of the stimuli from Experiment 1. Like in Experiment 1, occupations were used for person words, while location words were selected to be used with the preposition *in*. Compound words were excluded if they were not highly lexicalized (e.g., *filmhuis* —cinema). In each list, any word had to have a higher pairwise similarity to any other word from the same group than for any other word of a different group. In other words, in‐group similarity, as measured by cosine similarity, was always greater than out‐group similarity. In addition to the inspection of the pairwise similarity comparisons, we visualized the embeddings using t‐distributed stochastic neighbor embeddings (t‐SNE, Van der Maaten & Hinton, 2008). Figs. 3 and 4 show the t‐SNE visualizations of the Word2vec embeddings of the selected person and location words. This visualization displays more similar embeddings closer together and, in return, more dissimilar embeddings further apart. In the figure, groups of words that are semantically related according to the pairwise similarity we calculated earlier are displayed in the same color and can be seen to mostly form distinct groups.

To create the new stimulus sets, the person and location words were combined according to the pattern used in Experiment 1. An example stimulus set consisting of 24 sentences can be seen in Table 4. Instead of sentences with repeated lexical items in Experiment 1 (e.g., “The captain is in the tower” and “The captain is in the swimming pool,” see Table 1), participants learned sentences with semantically related lexical items in Experiment 2 (e.g., “The captain is in the tower” and “The admiral is in the swimming pool”). Table 4 shows that the lexical items corresponding to the same letter in the pattern are semantically related, for example, *admiraal* and *kolonel*, which both refer to military personnel. The subscript numbers refer to the distinct items selected from the semantically similar word lists. Before the creation of each participant's unique stimulus set, the order of the lists within one category and the order of the items within one list were shuffled.

**Table 4:** A full set of target stimuli used in Experiment 2

Person	Location	Example sentences
$a_{1}$	$A_{1}$	*De admiraal is in de afgrond*
$b_{1}$	$A_{2}$	*De denker is in het ravijn*
$a_{2}$	$B_{1}$	*De kolonel is in de fabriek*
$c_{1}$	$B_{2}$	*De dokter is in de montagehal*
$b_{2}$	$C_{1}$	*De wetenschapper is in het appartement*
$d_{1}$	$C_{2}$	*De jager is in de woning*
$c_{2}$	$D_{1}$	*De internist is in het fort*
$d_{2}$	$D_{2}$	*De stroper is in het kasteel*
$e_{1}$	$E_{1}$	*De advocaat is in het plaatsje*
$f_{1}$	$E_{2}$	*De graficus is in het gehucht*
$e_{2}$	$F_{1}$	*De raadsman is in de faculteit*
$f_{2}$	$F_{2}$	*De schilder is in de universiteit*
$e_{3}$	$G_{1}$	*De notaris is in de synagoge*
$g_{1}$	$G_{2}$	*De artiest is in de tempel*
$e_{4}$	$H_{1}$	*De procureur is in het warenhuis*
$g_{2}$	$H_{2}$	*De zanger is in de supermarkt*
$f_{3}$	$I_{1}$	*De illustrator is in het woud*
$h_{1}$	$I_{2}$	*De monteur is in het bos*
$f_{4}$	$J_{1}$	*De kunstenaar is in het terras*
$h_{2}$	$J_{2}$	*De installateur is in de serre*
$g_{3}$	$K_{1}$	*De muzikant is in de graafmachine*
$h_{3}$	$K_{2}$	*De elektricien is in de kiepwagen*
$g_{4}$	$L_{1}$	*De deejay is in de hut*
$h_{4}$	$L_{2}$	*De loodgieter is in de tent*
Re‐paired foils:		*De advocaat is in de afgrond*
		*De raadsman is in de fabriek*

*Note*: In this example, person fan is varied (e.g., a = fan 2, e = fan 4), while location fan remains at fan 2. The letters refer to the lists from which the items are selected. $a_{1}$ and $a_{2}$ are selected from the same list of words, but are different lexical items in this list.

#### 4.1.2 Participants, design, and procedure

We used the PsychoPy script of Experiment 1 in this experiment. We collected data from adult Dutch L1 speakers (*N* = 100) from the Netherlands and Belgium online through Prolific with high approval ratings (95–100%). All participants provided informed consent. The experiment consisted of the same exposure, learning, and testing phase as in Experiment 1 and took participants about 30 min to complete.

### 4.2 Results

Due to the overall high accuracy in the data from this experiment, we excluded participants with an accuracy below 0.65, resulting in 97 participants in the final data set. The models we used for the analysis of the data were identical to the ones used in Experiment 1.

#### 4.2.1 Accuracy

The mean accuracies recorded during the retrieval task in the testing phase are presented in Table 5. Overall, participants performed the task with high accuracy, with responses to sentences in which both concepts were at fan 2 being only marginally more accurate than sentences in which one concept was at fan 4. The increase in accuracy in this experiment as compared to Experiment 1 may be due to the change in stimulus sentences to use unique instead of repeated lexical items, which may have facilitated the retrieval of specific sentences.

**Table 5:** Descriptive summary of mean accuracies with standard errors (SE) in Experiment 2

Person fan	Location fan	Accuracy (SE)
2	2	0.93 (0.004)
2	4	0.92 (0.004)
4	2	0.92 (0.004)

**Fig. 5:**
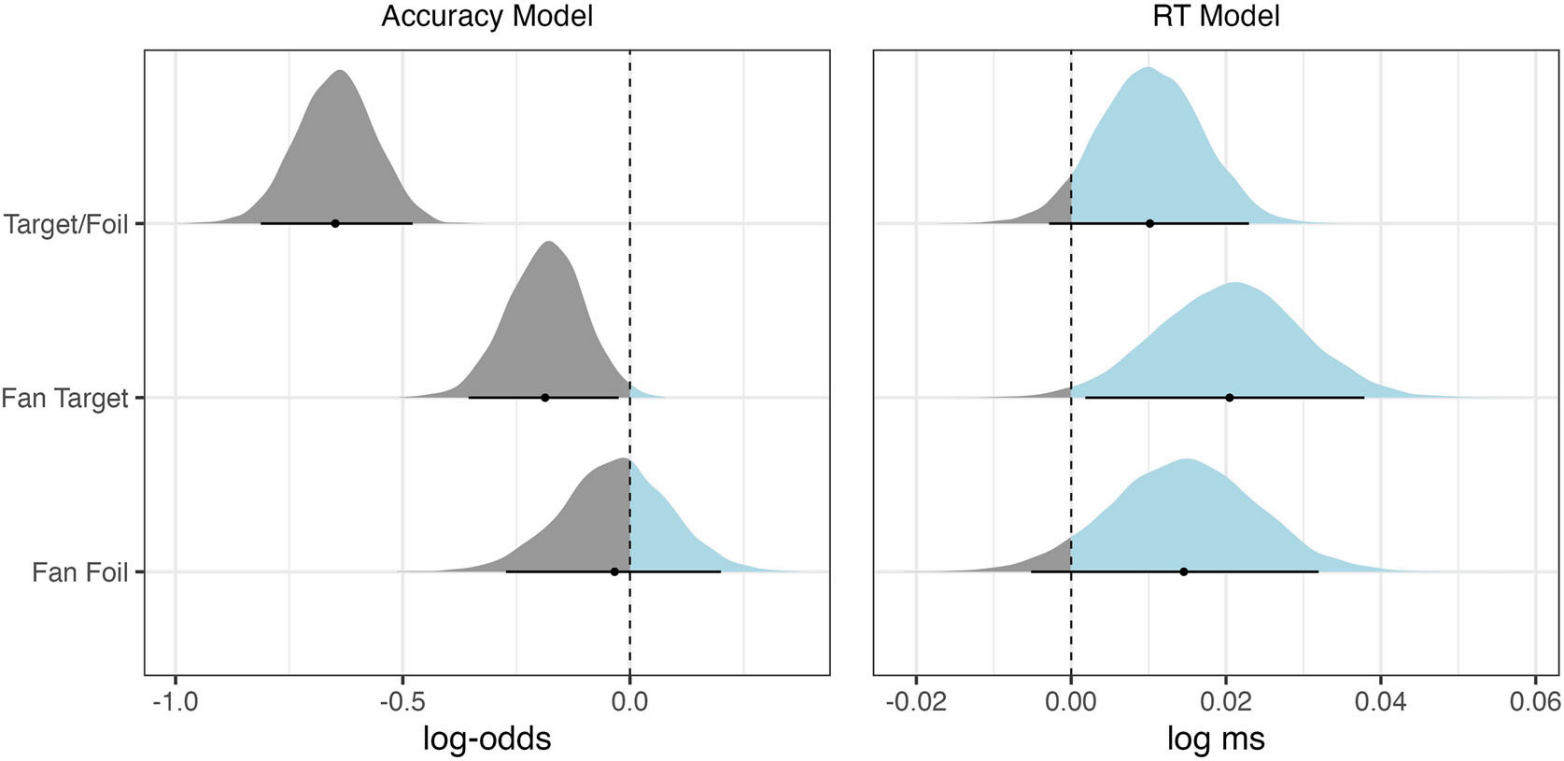
Posterior distributions of the accuracy model (left) and the reaction time model (right) fit on data collected in Experiment 2.

We fitted the same Bayesian model to these data as we did in Experiment 1. The model converged with reported Rhat values close to 1 (max. Rhat = 1.004) and no divergent transitions after warm‐up. The posterior distributions are shown in the left plot of Fig. 5. The log‐odds of Fan Target (*fan_target*) are largely negative and the 95% CI, indicated by the black line underneath the distribution, does not cross 0. This indicates that an increase in fan from 2 to 4 decreased the accuracy with which participants identified target sentences. The posterior distribution for Fan Foil (*fan_foil*) has a mean that is close to 0, which means that we cannot observe a fan effect for foils in the accuracy measure. The fan effect on foils, however, was not the objective of this experiment and a similar result was found for accuracy measures on foils in the lab data of Experiment 1. The posterior distribution of Target/Foil (*stimulus_type*) is negative, showing that participants were less accurate when identifying foil sentences than they were for target sentences.

#### 4.2.2 Reaction times

Before fitting the Bayesian model to the RT data, we cleaned the data by removing extreme RTs smaller than 200 ms and larger than 1 min and 30 s, resulting in the removal of 64 data points (0.4% of the data). Afterward, we removed RTs that were outside of three standard deviations from the mean of the RT data. The mean RTs collected in the testing phase of the experiment are presented in Table 6. The general range of RTs is similar to that of RTs from the online version of Experiment 1 and an overall increase can be seen for fan 4 in both person and location conditions. Note that, as in Experiment 1, fan 2 values are identical across the person, location, and combined columns. This reflects the design, where one category was held at fan 2, while the other varied, so the subset of trials for fan 2 is the same across person and location. These values are not independent observations.

**Table 6:** Descriptive summary of mean reaction times (in ms) with standard errors (SE) in the data collection of Experiment 2

Condition	Fan	RT (SE)
person	2	1854 (27.9)
	4	1918 (27.2)
location	2	1854 (27.9)
	4	1904 (28.9)
combined	2	1854 (27.9)
	4	1951 (19.3)

To model RTs, we fitted the same model to the data as in Experiment 1. The posterior distributions of the RT model are presented in the right plot of Fig. 5. For both Fan Target and Fan Foil, the distributions lean toward positive values, showing that an increase of fan from 2 to 4 leads to longer RTs in both conditions. The 95% CIs of Fan Foil includes 0, meaning that this effect is more uncertain. Moreover, the posterior distributions of Fan Target and Fan Foil have a wider spread compared to Experiment 1, indicating a wider range of RTs in response to both target and foil sentences. The posterior distribution of Target/Foil includes largely positive values, indicating that participants showed larger RTs for targets than for foils. The 95% CI of this distribution also includes 0, indicating some uncertainty about the effect. Interestingly, this effect differs from Experiment 1, where targets were reacted to more quickly than foils.

Finally, we explore the evidence for the fan effect using the Bayes factor analysis, in which we compare the M_1_ model, which includes the fan contrast nested in the target condition, with the baseline (M_0_) model in which the fan contrast nested in the target condition is missing in fixed effects. The random‐effect structure is identical for both models. The models are parallel to M_1_ and M_0_ models in the Bayes factor analysis of Experiment 1, but obviously excluding the data collection parameter.

We consider two prior distributions for the fixed factor *fan_target*: first, the prior is HalfNormal(0, 0.025) (the prior used for the Bayes factor analysis in Experiment 1); second, the prior is Normal(0.026, 0.01) (the posterior of fan_target found in Experiment 1). For the first prior, we find, using the Savage–Dickey ratio method, that ${\text{BF}}_{10}$ is 4.3. For the second prior, we see that ${\text{BF}}_{10}$ is 7.1. Both values reveal moderate evidence in favor of the model, which includes the fan fixed effect for targets, relative to the model that lacks the fan fixed effect for targets.

### 4.3 Discussion

**Fig. 6:**
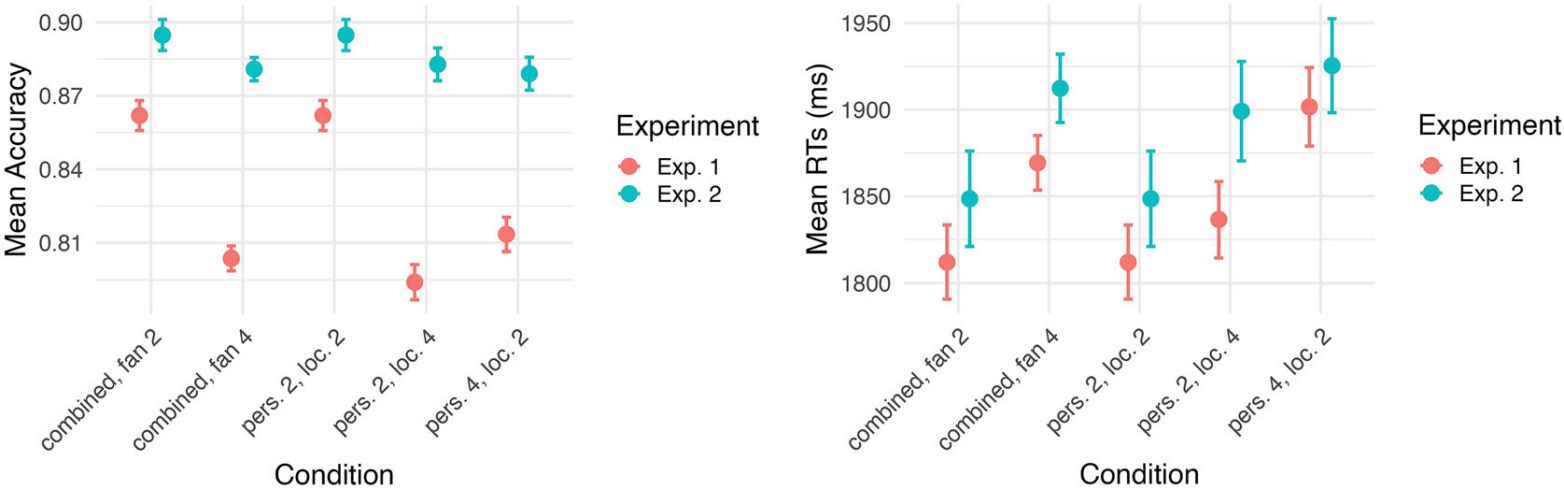
Mean accuracy (left) and RTs (right) over all experimental conditions in both experiments.

The results of Experiment 2 show that the fan effect persists when we create the associative fan with semantically related items instead of using items that occurred repeatedly in different stimulus sentences. We could detect the fan effect in accuracy and RTs of target sentences: participants responded to higher fan conditions with lower accuracy and higher RTs. In both Experiment 1 and Experiment 2, we found a clear fan effect: as the number of associations to a concept increased, participants became slower and less accurate in retrieving the related context. Fig. 6 summarizes the two results of the two experiments. In Experiment 1 (red), we see that the mean accuracy decreased for fan 4 conditions (left panel), while mean RTs increased for higher fan conditions (right panel). We observe the same pattern in Experiment 2 (blue). This shows that even when associations were based on semantic similarity rather than repeated lexical items, the fan effect can be observed in a small drop in accuracy and an increase in RTs for the fan 4 condition.

Interestingly, Experiment 1 and Experiment 2 also differ, in particular, with regard to the performance on targets and foils. In Experiment 1, participants identified targets more easily than foils which is reflected in faster RTs and higher accuracy in identifying targets. In Experiment 2, however, foils were identified more easily than targets (faster RTs, higher accuracy). We hypothesize that in the classic fan paradigm in Experiment 1, the repeated use of lexical items across multiple sentences increased the base‐level activation of their representation in memory. While spreading activation was divided among multiple associations due to the fan condition, the familiarity and repeated exposure to the target items made the retrieval of target sentences from memory easier. In Experiment 2, however, the lexical items only occurred once, resulting in lower base‐level activation. This increased the difficulty of retrieving the target sentences from memory and allowed foils to be rejected more easily. These findings suggest that when base‐level activation is low and fan effects are created through semantic similarity rather than the repetition of an item, retrieval may be driven less by familiarity with an item and more by the distinctiveness of the specific combinations.

## 5 Representing the fan effects in vector‐space models of meaning

In Experiment 1, we showed that the classical fan effect exists in Dutch even if knowledge of the fan is acquired without explicit instruction about the fan size during the learning phase. In Experiment 2, we observed that semantic similarity, as measured in vector space models, also results in a fan effect.

In this section, we show in detail that vector‐space models of meaning can represent fan effects of both experiments. Focusing on the skip‐gram, for which we established equivalence between the dot products of word vectors and spreading activation in Section 2, we show that the model trained on Experiment 1 stimuli correctly predicts the fan 2 versus fan 4 contrast. We then show that the same model, fine‐tuned on pretrained embeddings, also predicts the fan effect due to semantic similarity in Experiment 2, which is impossible to capture for memory models that do not encode lexical knowledge. Injecting such lexical knowledge also allows ACT‐R to predict the fan effect of Experiment 2, albeit with a worse fit than the skip‐gram model. Finally, we show that dot products for individual word pairs predict RT variance beyond the coarse fan 2/fan 4 split.

### 5.1 Model specification

We train the skip‐gram model with five negative samples and noise distribution for negative samples set at $p^{3/4}$ (the default value, see also Mikolov et al., 2013).^2^ Our goal is to show that such a model will show the fan effect of Experiment 1, if trained from scratch, and it will show the semantic fan effect of Experiment 2, if fine‐tuned on pretrained lexical embeddings. This simulation thus complements our theoretical claim about the link between skip‐gram models and ACT‐R spreading activation memory, as discussed in Section 2.

Recall that the objective of skip‐gram models is to predict the context word(s) given the target word. For the purposes of the modeling of our experiments, we make the skip‐gram model predict only the words that could differ between items. Consider the example (5). If this is a sentence in the set in which the location fan was manipulated, then we will treat the location word (*toren* “tower”) as the target word. The person word (*kapitein* “captain”) is considered the context word, and the article accompanying the target word (e.g., *de* “the”) in (5) is another context word.^3^

(5) $\begin{array}{c} \text{De}\;\text{kapitein}\;\text{is}\;\text{in}\;\text{de}\;\text{toren}.\\ \text{The}\;\text{captain}\;\text{is}\;\text{in}\;\text{the}\;\text{tower}. \end{array}$

No other words are included in the model, since they were identical for all items and consequently, adding them as extra target–context word pairs would not affect the outcome of the model.

At the start of the training for Experiment 1, we initialized the target and context words by randomly sampling each vector value from uniform(−1, 1). The random initialization reflects the assumption, made by Anderson and Reder (1999) and others, that the person and location words during the fan experiment are used as mere memory placeholders. For Experiment 2, we used Dutch word2vec pretrained embeddings from Fares et al. (2017) for target words, that is, for person words if the person fan was manipulated and location words if the location fan was manipulated. The context words were initialized by random sampling from uniform(−1, 1). All vectors were of size 100 dimensions, which was the dimension of the pretrained embeddings used in modeling Experiment 2.^4^

Training was performed using adaptive backpropagation as implemented in the Adam optimizer, with lr = 0.003. The training updated the vectors for both target and context words in the skip‐gram model for Experiment 1. For Experiment 2, only the context word vectors were updated (the pretrained embeddings were frozen during our training stage).

We trained the model with three different seeds and averaged over the models. Training was done until the model learned the task close to perfectly (1% or less of mistakes in recall of the target–context pairs). This required 24 training epochs in Experiment 1 and 65 training epochs in Experiment 2. For Experiment 1, only one stimuli list was used since particular lexical specifications (i.e., how persons and locations are related) did not matter for the model in which all vectors were initialized randomly. For Experiment 2, we trained 100 separate skip‐gram models, one for each stimulus list.

To be clear, our goal is not to show that skip‐gram models can learn the fan experiment perfectly or almost perfectly. This is to be expected. However, we do claim that the resulting model is a good approximation of human memory, and we explore and show that below.

### 5.2 Modeling results

As we have seen in Section 2, the skip‐gram model is related to pmi, which in turn is spreading activation according to the rational analysis of memory. To approximate pmi in the skip‐gram model using the target and context vector, the following equation applies, where k is the number of negative samples, t is the target word, c is the context word, and t and c are target and context word vector representations in the model (see Levy & Goldberg, 2014 for details).

(6) pmi(t,c)≈t·c+logk

We now use this link to connect the trained skip‐gram models and our findings in Experiment 1 and Experiment 2.

#### 5.2.1 Experiment 1

We first focus on the difference between activations for fan 2 and fan 4 in Experiment 1. After our training of the randomly initialized vectors is done, we collect pmi from the models, following (6). We calculate the dot product for all target–context word pairs and shift by +log(5) (since the model used five negative samples). The calculation is the same for fan 2 and fan 4 cases. If we are right about the link between the skip‐gram vector model and the rational theory of memory, the values should represent spreading activations in the ACT‐R memory model.

We indeed find that the dot products of the two cases differ in the expected way. We see that the average shifted dot product for fan 2 is higher (mean = 1.73, sd = 0.15) than for fan 4 (mean = 1.22, sd = 0.06). Following ACT‐R, the shifted dot product can be linked to behavioral measures as shown in (7). These are the standard linking functions from ACT‐R (Anderson, 2007), with F a scaling parameter, T the retrieval threshold, and s noise. $A_{i}$ is the (spreading) activation of a chunk, represented, in our case, by the shifted dot product of word pairs. It follows from this that fan 2 items are predicted to take less time and have higher accuracy than fan 4 items. This is in line with our experimental findings.

(7) $\begin{array}{c} a.\;\text{Retrieval}\;\text{time}\;\text{of}\;\text{chunk}\;i:T_{i}=Fe^{-A_{i}}\\ b.\;\text{Probability}\;\text{of}\;\text{retrieval}\;\text{of}\;\text{chunk}\;i:P_{i}=\frac{1}{1+e^{-(A_{i}-T)}/s} \end{array}$

The correct prediction has been reached in several reasoning steps: (i) we observed a formal link between the spreading activation model of memory and skip‐gram models; (ii) we trained the skip‐gram models and collected the pmi values from them, which represent spreading activations; (iii) we assumed the standard linking function between the activations and behavioral measures of accuracy and retrieval times from ACT‐R.

#### 5.2.2 Experiment 2

Since we have now established the link between skip‐gram and spreading activation and confirmed it for Experiment 1, we can take our modeling one step further and capture the effect of semantic similarity in Experiment 2. Recall that for this modeling, we use pretrained vectors as target vectors, which represent lexical knowledge, and we randomly initialize context vectors, and we train the latter on the fan experiment to hopefully derive the semantic fan effect.

First, we check that the fan effect is not present in the stimuli lists *before* any training, that is, just in pretrained embeddings. We see that the average dot products for fan 2 and fan 4 show virtually no difference (for the location fan: fan 2: mean 1.64, sd = 0.44; fan 4: mean = 1.65, sd = 0.29; for the person fan: fan 2: mean = 1.67, sd = 0.40; fan 4: mean = 1.65, sd = 0.25). We can be confident that we did not inadvertently create stimuli lists in which fan 2 elements had higher activations than fan 4 elements already in pretraining.

After the training, in which the model updated the context word vectors to learn the target–context word pairings in the fan experiment, we observe a clear change in the dot product values. The average dot product was 2.96 for fan 2 and 2.79 for fan 4 (for the location fan: fan 2: mean = 2.97, sd = 0.09; fan 4: mean = 2.81, sd = 0.06; for the person fan: fan 2: mean = 2.95, sd = 0.12; fan 4: mean = 2.78, sd = 0.06). That is, the skip‐gram model shows higher spreading activation for fan 2 compared to fan 4 after training on the experimental stimuli, which, per (7), predicts increased reaction times and decreased accuracy for fan 4 compared to fan 2 in Experiment 2. The difference in fan 2 and fan 4 is smaller than in Experiment 1, which goes in line with the fact that the difference in reaction times and accuracy is smaller than in Experiment 1.

To explore the predictions of the model further, we collect skip‐gram dot products for individual target–context word pairs, for all stimuli lists that were used in the experiment. We explore whether the values for individual word pairs can be used directly to predict RTs in Experiment 2 and how they compare to the model which simply encoded the 2–4 fan manipulation.

**Fig. 7:**
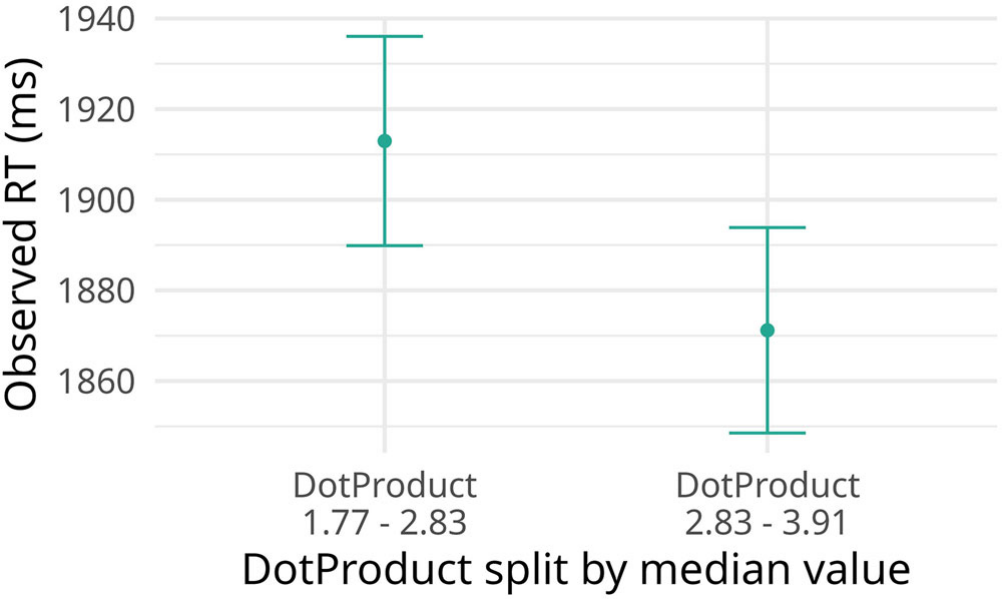
Mean and SE RTs for Experiment 2 split by dot product values. 2.83 is the median dot product value, the left and right bars show mean and SE RTs for those word pairs whose dot product is smaller than the median and greater than the median, respectively.

To understand the role of dot product on RTs, we subset the target data (i.e., exclude foils). For these data, we first check that when split into two groups by dot product values, smaller dot products align with higher RTs, see Fig. 7. Next, we construct a hierarchical Bayesian model with a shifted log‐normal likelihood, fitted to RTs, and investigate evidence for this model using the Bayes factor analysis. The model has one fixed effect: *dot_product*, collected from the skip‐gram model as described above, by subject random intercepts, and by word random intercepts and dot‐product slopes. We use HalfNormal(0, 0.025), just as in previous BF analyses, but this time on the negative side, reflecting the expectation that a higher dot product decreases RT. We keep the prior structure of the other parameters as in the reaction time models described in Section 3. We calculate evidence in favor of this model ($M_{1}$) compared to the baseline ($M_{0}$), which is the same but lacks the *dot_product* fixed effect. We see ${\text{BF}}_{10}$ of 10.7, which is moderate‐to‐strong evidence in favor of the model including the dot product. This evidence in favor of the dot‐product model is higher than the Bayes factor of the model with the fan parameter (${\text{BF}}_{10}=4.3$), presented in Section 4.

To explore further how the dot product compares to the fan 2/4 contrast, we consider a model with the shifted log‐normal likelihood and two fixed effects: *dot product*, collected from the skip‐gram model as described above, and *fan_target*, with contrast coding as +1 and −1 for target fan 4 and fan 2, respectively. Using the formula notation in R, we can specify the model as:

(8) $\begin{array}{ccc} \text{RT} & ∼ & 1+\text{dot}\_\text{product}+\text{fan}\_\text{target}+\\ & & +\;(1+\text{fan}\_\text{target}|\text{pp}\_\text{num})+\\ & & +\;(1+\text{dotproduct}+\text{fan}\_\text{target}|\text{fan}\_\text{word}) \end{array}$

**Fig. 8:**
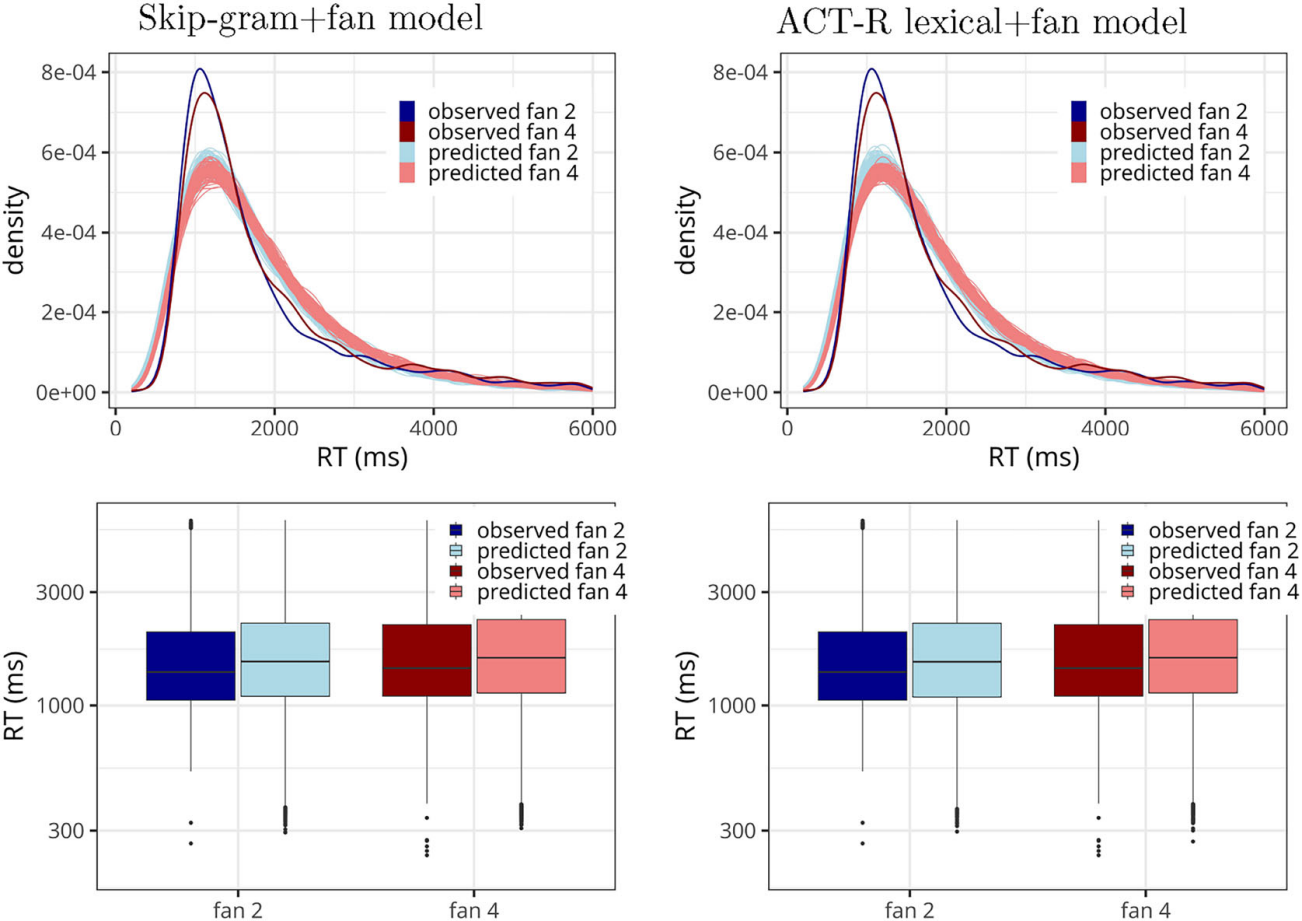
Posterior predictive checks showing RT distributions and box plots, split by fan size. Note that the y‐axis of the box plots is log‐scaled. The left side: The skip‐gram+fan model, see the formula in (8). The right side: The ACT‐R+fan model, see the formula in (12).

**Fig. 9:**
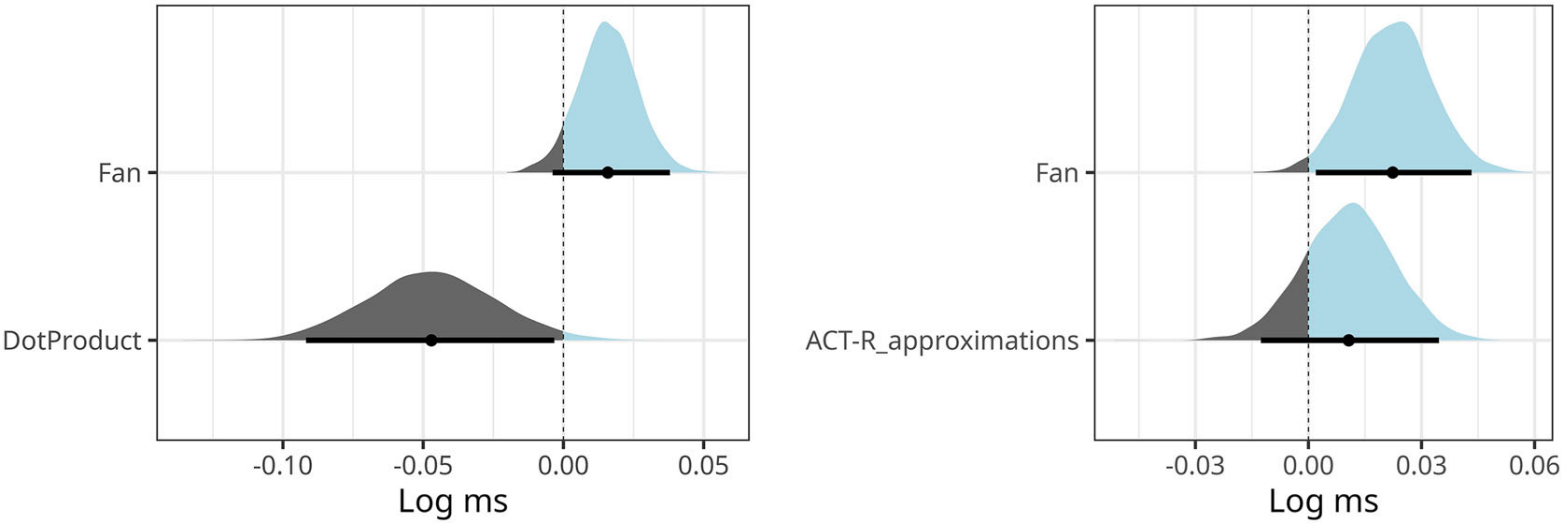
Posterior distributions of the model using the skip‐gram dot product (left) and the model approximations using discretized values in word vectors for ACT‐R estimations of spreading activation (right) in Experiment 2.

The prior structure of the model was the same as in the other Bayesian models (see Section 3). The posterior predictive checks, visualized on the left side in Fig. 8, reveal that the model approximates the RT distribution reasonably well. The posterior distributions of the fixed effects are summarized in the left graph in Fig. 9 and reveal the expected negative effect of *dot_product*, whose 95% CI moreover excludes zero (mean = −0.047, 95% CI = [−0.091, −0.002]).

The comparison of the role of *dot_product* and *fan_target* is further studied using a Bayes factor analysis. We contrast $M_{\text{dp}}$ (word‐pair *dot_product* as the fixed effect) with $M_{\text{fan}\_target}$ (*fan_target* as the fixed effect). Both models have the same intercept‐only random effect structure. We assume that the fixed effects follow the prior Normal(0, 0.1). We collect 10,000 samples for both models (with 5000 samples as warm‐up) and estimate Bayes factors ${\text{BF}}_{\text{dp,fan}}$ using bridge sampling to see the relative evidence that data provide for $M_{\text{dp}}$ over $M_{\text{fan}}$. ${\text{BF}}_{\text{dp,fan}}$ is 2.9, which, according to Jeffreys' interpretation of Bayes factor values, is on the borderline between barely worth mentioning and moderate evidence in favor of $M_{\text{dp}}$. Thus, we see some weak evidence for the dot‐product model and against the model that only encodes the fan similarity as a 2–4 contrast.

The amount of evidence might not seem like much, but we should keep in mind that the evidence in favor of the dot‐product model over the fan model is found in the experiment that was carefully designed to just study the fan effect and minimize variances in semantic similarity beyond the fan 2–fan 4 contrast. It is also good to remember that aside from this, we saw moderate‐to‐strong evidence for the model with the dot product over the baseline (intercept‐only) model; whatever evidence in favor of $M_{\text{dp}}$ is found in the 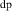
–$M_{\text{fan}}$ comparison, it has to be due to the effect of semantic similarity on reaction times that goes above and beyond the fan manipulation in the experiment.

#### 5.2.3 Modeling Experiment 2 by injecting the lexical knowledge into ACT‐R

We consider one last exploration of the connection between spreading activation in ACT‐R and skip‐gram models. In particular, we explore how the lexical knowledge of skip‐gram could be directly injected into an ACT‐R model. To this end, we take the pretrained word embeddings from the skip‐gram model that we used to model the fan effect of Experiment 2. Since ACT‐R works, at least as far as retrieval due to spreading activation is concerned, with only discrete cues—in our case, either match or mismatch of the cue—we need to binarize the values.^5^ We do so by using the sign function element‐wise on the vectors (i.e., for every dimension in the vector, if its value was positive, we code it as +1, otherwise as −1). Then, we calculate spreading activation using the ACT‐R formula for spreading activation (Anderson & Lebiere, 1998), (9). Here, S is the log size of memory, often set as a free parameter at some large positive number, to ensure that spreading activation will always be non‐negative.

(9) $S_{ji}=S-\log\left({\mathrm{fan}}_{j}\right)$

This equation reflects the spreading activation as approximation of pmi (see (1)), repeated in (10). Concretely, P(i), the probability of needing the chunk i, is commonly taken to be the reciprocal of the number of pieces of information that our memory holds, see (11[a]). The formula in (11[a]) provides the probability of needing the chunk i under the assumption that every piece of information is equally likely needed. The memory size is often not empirically found, but set as a free parameter, S. The numerator, P(i|j), is shown in (11[b]), where fan 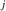 is the number of pieces of information that carry the cue j. (11[c]) shows the derivation of (9) from pmi under these assumptions.

(10) $S_{ji}=\log\frac{P(i|j)}{P(i)}$
(11) $\begin{array}{c} a.\;\;P\left(i\right)=\frac{1}{memory\_size}\\ b.\;\;P\left(i|j\right)=\frac{1}{{fan}_{j}}\\ c.\;\;S_{ji}=\log\frac{P(i|j)}{P(i)}=\log\frac{memory\_size}{{fan}_{j}}=\log\left(memory\_size\right)-\log\left({fan}_{j}\right)\\ \;=S-\log({fan}_{j}) \end{array}$

In our modeling of the semantic similarity experiment in ACT‐R, we treat every dimension as a cue, which results in 100 cues per word. As expected, we observe differences in ACT‐R activations for different fans in Experiment 2: the location fan: fan 2: mean 128.6, sd = 1.6; fan 4: mean = 125.2, sd = 1.1; the person fan: fan 2: mean = 125.4, sd = 1.8; fan 4: mean = 122.2, sd = 1.1. The higher activation values for fan 2 show that injecting the lexical knowledge into ACT‐R indeed also captures spreading activation effects due to semantic similarity, in particular, the fan 2/fan 4 contrast.

To explore the effects further, we calculate ACT‐R spreading activations per word, using the discretized values, and we use the estimates in a Bayesian model with a shifted log‐normal likelihood, fitted to RTs. The model has two fixed effects: ACT‐R spreading activation and fan manipulation, with the same random‐effects structure as in (12). This model thus parallels the model that we used to explore dot product effects on RTs. Using the R formula notation, we can unpack the model as:

(12) $\begin{array}{ccc} \text{RT} & ∼ & 1+\text{ACT-R approximation}+\text{fan}\_\text{target}+\\ & & +\;(1+\text{fan}\_\text{target}|\text{pp}\_\text{num})+\\ & & +\;(1+\text{ACT-R approximation}+\text{fan}\_\text{target}|\text{fan}\_\text{word}) \end{array}$

The posterior predictive checks, visualized on the right side in Fig. 8, show that the ACT‐R model fits the RT distribution comparably well to the skip‐gram model.

We are mainly interested in the posterior distributions of the fixed effects, which are summarized in the right graph of Fig. 9. The figure shows that the effect of ACT‐R activations, informed by skip‐gram vectors, does not add above and beyond the fan manipulation. Arguably, this is because we discretized the values, which led to information loss regarding semantic similarity. The comparison between the two graphs in Fig. 9 suggests that linking the spreading activation directly to the dot product is preferable over this method, which loses information. Finally, we also ran the Bayes factor analysis using bridge sampling to compare $M_{\text{dp}}$, presented in the previous section, with $M_{\text{actr}}$ (the model using ACT‐R activations of discretized values, and with the same intercept‐only random effect structure and the same prior for the fixed effects as $M_{\text{dp}}$ had). This comparison reveals strong evidence for $M_{\text{dp}}$: ${\text{BF}}_{\text{dp,actr}}=69$. Thus, while injecting the lexical knowledge into ACT‐R is possible and may be sufficient to capture the binary fan 2/fan 4 contrast in Experiment 2, there is evidence in favor of directly using the formal link between dot products of skip‐gram models and spreading activation, rather than approximating the vector‐space model as we did in this section.

## 6 General discussion

In this study, we established a formal link between spreading activation in models of human memory and semantic similarity in vector‐space models. Furthermore, we replicated the classical fan effect (see Anderson, 1974; Anderson & Reder, 1999) in Experiment 1 and significantly extended this paradigm in Experiment 2, which showed that the fan effect can also be found when spreading activation is triggered by semantic similarity.

We interpret the findings of this novel experiment as showing that the fan effect generalizes beyond tasks where the words are identical. Our Experiment 1 and previous studies can be seen as an extreme case of studying the role of semantic similarity on recall, one in which only the highest possible semantic similarity, namely, identity, is considered. As the results of Experiment 2 show, the fan effect is still present beyond this extreme case, in situations in which words within the same fan have “only” high semantic similarity but are not identical.

Our main findings can thus be summarized as follows. We provided evidence that cues in spreading activation, as tested in fan experiments, are not just concepts themselves, but unobservable sets of semantic features that the concepts share. Our theoretical and computational modeling results show that vector spaces, which measure affinity of concepts to those features, are particularly suitable to capture this more general view on spreading activation.

Our results show that, by successfully predicting behavioral data, vector‐space models have psychological validity in experiments centered around human language use. Our findings support the direct and theoretically justified connection between distributional semantic models and ACT‐R, as previously hypothesized by Hollis (2017) and discussed more broadly by Günther et al. (2019). Hollis suggested that the learning algorithms used in the continuous bag of words (CBOW) models (Mikolov, Chen, Corrado, & Dean, 2013) approximate the retrieval mechanisms in ACT‐R, specifically through the concept of need probability, showing that the estimates based on CBOW correlate with behavioral measures of lexical decision and naming times. Our study takes this argument one step further by aligning the structure of skip‐gram embeddings directly with the rational analysis of memory, underpinning ACT‐R's spreading activation mechanism using the fan effect. This allows us to test more directly how semantic similarity shapes memory activation within the ACT‐R framework. This approach is further supported by Günther et al. (2019), who argue that prediction‐based models such as skip‐gram, when they integrate measures like pmi, are cognitively plausible representations of meaning because they capture contextual dependencies. Our results contribute to a growing body of work, suggesting that semantic similarity affects memory retrieval in language (Jäger et al., 2017; Laurinavichyute & von der Malsburg, 2022; Smith & Vasishth, 2020; Van Dyke, 2007). Using vector‐based models has also been successful in other memory frameworks, like MINERVA2 (Hintzman, 1984), which can capture various false recognition findings when combined with word2vec models (Chang & Johns, 2023).

Additionally, our findings show that fan effects can be observed in Dutch, adding to previous literature, which demonstrated that the fan effect is not specific to a particular linguistic context (aside from English, it is experimentally confirmed in Spanish, Gómez‐Ariza & Bajo, 2003; Japanese, Hirai, Hiwatashi, Kikuchi, & Kamijo, 1987; and German, Rösler, Heil, & Glowalla, 1993).

There have been other attempts in the past to connect ACT‐R and vector‐space models. In psycholinguistics, Smith and Vasishth (2020) and Meghdadi et al. (2026) combined the two to study lexical and sentence processing. In cognitive science, the holographic declarative memory model (Kelly, Arora, West, & Reitter, 2020) was used for modeling fan effects, among others. Unlike our analysis, these approaches substitute the existing memory module of ACT‐R, in particular spreading activation, with a completely novel account. In contrast to that, we showed how one can make use of the underlying link between rational analysis and pmi, on one hand, and pmi and vector dot products of skip‐gram models, on the other hand, to formally and in a principled manner link rational analysis and vector‐space models. In short, we do not have to enrich the memory module with new properties and parameters. Instead, there is a good reason to simply see the skip‐gram model as yet another implementation of the rational analysis of memory, one that can supply rich spreading activation between all lexical items.

Intuitively, one obvious reason why frameworks without rich semantic spaces, like the classical ACT‐R, are often enriched is arguably the fact that these models work with too coarse‐grained cues. This limits their ability to predict fan effects beyond anything else than the word identity. The skip‐gram model, on the other hand, decomposes a word into a high‐dimensional vector and the fine‐grained representation allows it to capture fan effects due to word identity *and* fan effects due to word similarity. As we also saw, using more fine‐grained cues in ACT‐R does improve the model's prediction and does allow the ACT‐R model to capture the contrast in fan in Experiment 2. However, using skip‐gram vectors with continuous values and their dot products as an estimate of spreading activation is theoretically justified and it seems a better representation, as it also explains RT variance in Experiment 2.

A key part of our study is the use of pmi as it fits mathematically into the ACT‐R model and offers a more interpretable and direct measure of semantic similarity. Importantly, pmi has previously been shown to perform as well as or better than, for example, latent semantic analysis (LSA, Landauer & Dumais, 1997) in capturing semantic similarity across different tasks (Turney, 2001; Van Maanen et al., 2010).

We should note that there are also several other explanations for the fan effect, which do not assume the effect arises through cue sharing in spreading activation. These explanations range from typicality (Silber & Fisher, 1989), through the plausibility of situation models (Radvansky & Zacks, 1991) to memory inhibition (Anderson & Spellman, 1995). It remains to be seen whether these accounts could be extended to our fan‐effect findings in Experiment 2. This particularly concerns the plausibility of situation models, which argue that a higher fan leads to less plausible situations. This is because, in the classic fan paradigm, a fan higher than one represents a situation in which one person is in multiple locations at the same time (or one location hosts multiple people at the same time). Especially the former, possibly even the latter becomes less plausible as fan size increases. In our Experiment 2, we find the fan effect even though we used unique person–location pairs throughout the experiment. As far as we can see, this finding cannot be predicted by plausibility of situation models.

A limitation of our study is that while we used nearest neighbors from vector‐space models to create groups of semantically related items for Experiment 2, the fan manipulation remained to some extent artificial. The groupings were constrained to two fan levels (2 or 4), and in natural language use, concepts often have a wider and more variable range of associations, resulting in more salient manipulations, even implicitly. Additionally, the short and simple sentences used in our paradigm are simplified versions of the complex syntactic and contextual structures that language users encounter in natural language. However, it should be stressed that Experiment 2 is the first time natural variation in language is used to elicit fan effects. Moreover, we found support for the notion that semantic similarity, not the classical fan effect as used in Experiment 1, explained the behavioral effects in Experiment 2. This suggests that, despite the relatively artificial set up, semantic associations drive the fan effect.

An avenue for further research is the extension of this paradigm to large language models (LLMs). In our current study, we demonstrated that semantic representations derived from a skip‐gram model can be meaningfully linked to human memory. The skip‐gram model is grounded in a well‐understood mathematical framework, which provides a foundation for future work using more complex, context‐based embeddings from LLMs. However, unlike vector space models, LLMs operate in highly complex architectures in which internal mechanisms are more opaque. Wang et al. (2025) specifically address the comparability of semantic networks in LLMs and humans and find significant differences in terms of flexibility and interconnectivity, as well as local association organization based on the performance in a semantic fluency task. These structural differences may help explain the variability of fan effect results observed in LLMs as reported by Roberts et al. (2024), where some of the LLMs fail to exhibit the fan effect.

In sum, our study shows that the fan effect is observed in cases that go beyond just word identities, and that the research on memory retrieval in fan studies needs to take into account that retrieval cues should be semantic features. This, in turn, directly connects the rational analysis of memory with Natural Language Processing (NLP) research on decomposing the lexicon (see, e.g., Korchinski, Karkada, Bahri, & Wyart, 2025 for a recent theoretical account). Further research into the connection should be beneficial for both research areas.

## 7 Conclusion

This study examined the fan effect in Dutch and its extension to semantically similar stimuli generated from vector‐space models. In Experiment 1, we validated conducting the fan effect in Dutch with an adjusted learning method in which the fan manipulation was not made explicit to the participant. In Experiment 2, we extended this paradigm by creating the associative fan based on semantic similarity, demonstrating that the fan effect persists even when associations are inferred from natural language rather than learned explicitly. Taken together, our findings show that associative interference in memory is not limited to repeated stimuli but also emerges from semantic structure. This suggests that memory retrieval in language is sensitive to the underlying relationships between concepts to be retrieved, even when these relationships are not explicitly learned. Future studies could explore how such interference affects memory retrieval in more complex linguistic contexts and how it interacts with factors such as discourse structure, individual differences in memory capacity, or predictability.

## Conflicts of interest

The authors declare no conflicts of interest.

## Permission to reproduce material

All materials used in this study are original.

## Data Availability

The data, materials, and analysis scripts used for this study are available on OSF: https://osf.io/5hw3n

## Notes

1 The posterior distributions in Figure 2 are shown on the log‐transformed RT scale. The descriptive statistics are provided in Table 3 to aid interpretation.

2 The code for the training of the models and for the analysis can be found on github: https://github.com/jakdot/fan‐similarity.git.

3 We include the article in modeling because it could vary across items—it could appear as *de* (common gender) for some items, and as *het* (neuter gender) for the rest, see also Section 3.

4 For Experiment 1, we also tried smaller vector dimensions (of size 30 and 50), but this did not affect the results.

5 An anonymous reviewer points out that the ACT‐R model also works with partial matching, which allows modelers to implement graded similarity penalties in retrieval. However, partial matching normally models the gradedness of the cue–chunk match, not spreading activation. It is the latter that is the relevant mechanism for capturing fan effects, as it distributes activation across competing memory chunks as a function of fan size.
